# Molecular and Functional Characterization of a Polygalacturonase-Inhibiting Protein from *Cynanchum komarovii* That Confers Fungal Resistance in Arabidopsis

**DOI:** 10.1371/journal.pone.0146959

**Published:** 2016-01-11

**Authors:** Nana Liu, Xiaowen Ma, Sihong Zhou, Ping Wang, Yun Sun, Xiancai Li, Yuxia Hou

**Affiliations:** College of Science, China Agricultural University, Beijing, 100193, China; The Chinese University of Hong Kong, HONG KONG

## Abstract

Compliance with ethical standards: This study did not involve human participants and animals, and the plant of interest is not an endangered species.

Polygalacturonase-inhibiting proteins (PGIPs) are leucine-rich repeat proteins that plants produce against polygalacturonase, a key virulence agent in pathogens. In this paper, we cloned and purified CkPGIP1, a gene product from *Cynanchum komarovii* that effectively inhibits polygalacturonases from *Botrytis cinerea* and *Rhizoctonia solani*. We found the expression of *CkPGIP1* to be induced in response to salicylic acid, wounding, and infection with *B*. *cinerea* and *R*. *solani*. In addition, transgenic overexpression in Arabidopsis enhanced resistance against *B*. *cinerea*. Furthermore, CkPGIP1 obtained from transgenic Arabidopsis inhibited the activity of *B*. *cinerea* and *R*. *solani* polygalacturonases by 62.7–66.4% and 56.5–60.2%, respectively. Docking studies indicated that the protein interacts strongly with the B1-sheet at the N-terminus of the *B*. *cinerea* polygalacturonase, and with the C-terminus of the polygalacturonase from *R*. *solani*. This study highlights the significance of CkPGIP1 in plant disease resistance, and its possible application to manage fungal pathogens.

## Introduction

The cell wall is the plant’s first line of defense against pathogens, most of which secrete degradative enzymes early in infection [1]. To prevent infection, plants express cell surface receptors that detect and identify pathogens, and trigger the appropriate defense response. One such receptor is polygalacturonase-inhibiting protein (PGIP), which specifically inhibits endopolygalacturonase, the first enzyme produced by invading pathogens to macerate host tissues [2, 3] and facilitate further degradation by other enzymes [4]. Therefore, polygalacturonase is a key virulence factor in fungi such as *Botrytis cinerea* [5, 6], *Aspergillus flavus* [7], and many others [8–10]. The enzyme is also required for infection by some *Phytophthora* pathogens like *P*. *capsici* [11].

PGIP expression is induced by many biotic or abiotic stimuli, including fungi, insects, mechanical damage, salicylic acid, methyl jasmonate, and oligogalacturonic acid [12]. For example, oligogalacturonic acid and mechanical damage boost expression of PGIP genes in *Phaseolus vulgaris* [13]. Komjanc et al. [14] also found that salicylic acid or infection with *Venturia inaequalis* triggers accumulation of PGIP mRNA. Nevertheless, the mechanism that regulates PGIP gene expression is unclear.

Notably, overexpression of pear PGIP enhances resistance against *B*. *cinerea* in tomato [15], *Arabidopsis thaliana* [16], and other plants [17–19]. Conversely, antisense expression in *A*. *thaliana* increases susceptibility to *B*. *cinerea* [20]. Taken together, these reports indicate that plant resistance to pathogens can be enhanced by transgenic expression of PGIP.

PGIP genes and isoforms have different specificities and inhibitory activities [21]. For example, both PGIP genes in *A*. *thaliana* inhibit *Colletotrichum acutatum* and *B*. *cinerea* [16, 22]. In contrast, only one of four PGIP genes in soybean is active [13]. In *P*. *vulgaris*, one PGIP gene is active against *Fusarium moniliforme* [23] and *A*. *niger*, while the other does not [24]. Remarkably, strains of the same species often have variable PGIP-mediated resistance against different pathogens [21].

Complexes between polygalacturonase and PGIP are considered a model system of plant-pathogen interactions [25]. However, most data have been obtained from studies of *P*. *vulgaris* PvPGIP2, the crystal structure of which is the only PGIP structure available [26, 27]. Fortunately, protein homology modeling and docking have enabled in-depth analyses of other enzyme-inhibitor complexes [28, 29].

*Cynanchum komarovii* Al Iljinski is a perennial, erect, or half-erect herbaceous plant that grows in desert and semi-fixed dunes in northwest China. It is traditionally used as an analgesic, antifungal, anti-inflammatory, and immunostimulatory agent, or to control agricultural pest and disease. The proteins CkTLP and CkChn134 from this plant have strong antifungal activity against *Rhizoctonia solani* and *B*. *cinerea* [30], which are important pathogens of tomato and rice, respectively.

In this paper, we assess the ability of CkPGIP1 from *C*. *komarovii* to confer fungal resistance in *A*. *thaliana*. We found that overexpression enhanced resistance to *B*. *cinerea*. In addition, purified recombinant CkPGIP1 significantly inhibited *B*. *cinerea* and *R*. *solani* polygalacturonase *in vitro*. Finally, protein docking methods indicated that CkPGIP1 interacts strongly with the N-terminal region of the polygalacturonase from *B*. *cinerea*. The inhibitor also binds the C-terminus of the enzyme from *R*. *solani*, but not with the same affinity. Docking analyses from another hand explained the *in vitro* results, and for better exploring the putative amino acids that involved in protein-protein interaction. These results enhanced our understanding of fungus-plant interactions via PGIP and polygalacturonase.

## Results

### Cloning and characterization

*CkPGIP1* cDNA was obtained by colony *in situ* hybridization (GenBank accession no. KP938429). The gene contains an open reading frame of 1014 bp, and encodes a protein of 338 amino acids with isoelectric point 8.81 (S1 Fig) and molecular weight 35.2 kDa. The coding sequence does not contain introns, although introns are present in homologs in peach and Arabidopsis [31].

**Fig 1:**
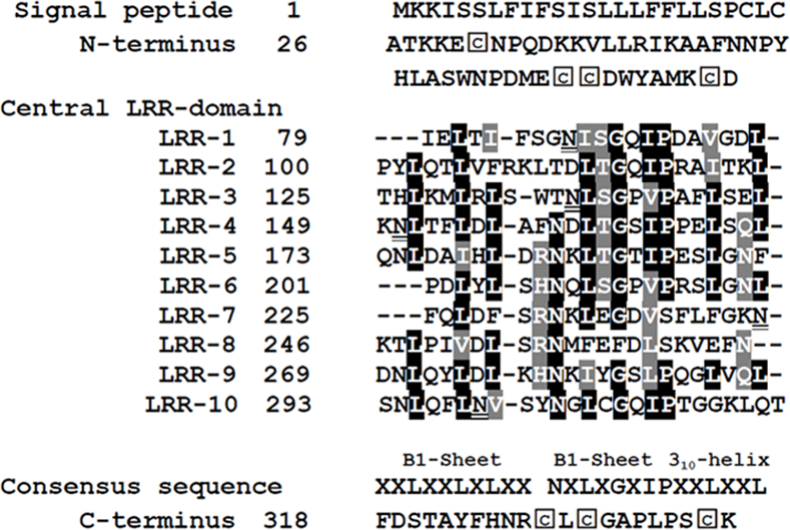
The PGIP-specific consensus sequence xxLxLxx.NxLx..GxIPxxLxxL.xxL in leucine-rich repeat units of CkPGIP1. Secondary structure elements (sheets B1, B2, and 3_10_-helix) are indicated for a homology model of CkPGIP1, which is based on PvPGIP2 (1OGQ) [26]. Putative glycosylation sites are doubly underlined, while conserved C residues are marked using diamonds.

We used the SMART program to analyze the domain structure of CkPGIP1 and its phylogenetic relationship to other PGIPs. A putative, functionally critical signal peptide [26] of 25 amino acids was detected (Fig 1). The main domain is comprised of 10 imperfect LRRs, each about 24 amino acids long, with the consensus sequence characteristic of PGIP. This sequence, xxL xLx x.N xLx..GxIPxxLxxL.xxL [26], is thought to mediate protein-protein interactions. Notably, CkPGIP1 contains the β-sheets B1 and B2, as well as the 3_10_-helix found in PvPGIP2. In PvPGIP2, the B1 sheet interacts intimately with the right-handed superhelix formed by LRR units. On the other hand, the B2 sheet is found only in PGIPs, but not in other LRR proteins [26].

**Fig 2:**
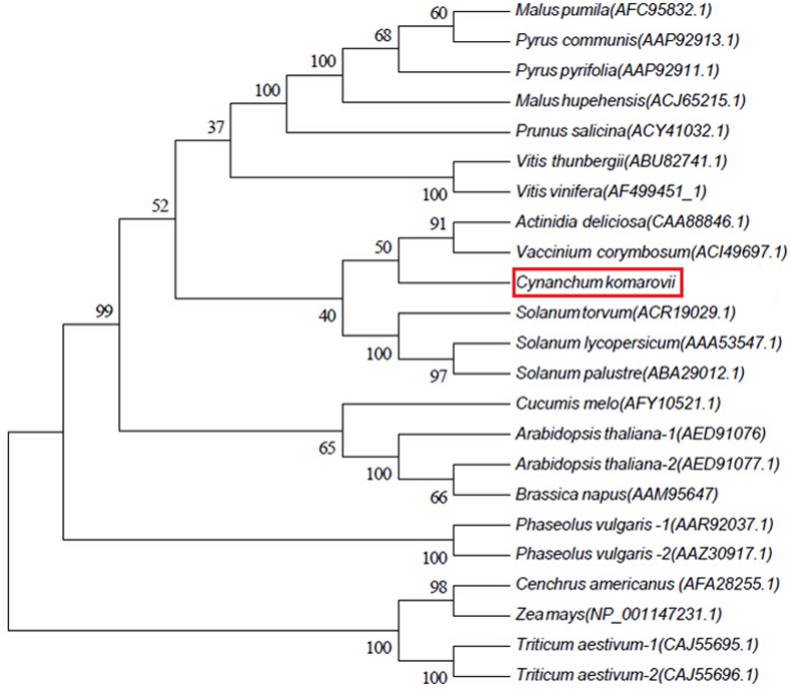
Phylogenetic analysis of CkPGIP1 and other known PGIPs. Amino acid sequences of CkPGIP1 (red box) and other known PGIPs were obtained from GenBank. The neighbor-joining tree was built in MEGA 5.1 based on a multiple sequence alignment.

As in PvPGIP2, the main domain in CkPGIP1 is flanked by N- and C-terminal regions with conserved C residues that form a disulfide bond, which is necessary for structural integrity [26]. Furthermore, five N-glycosylation sites were predicted by NetOGlyc 4.0, with consensus sequence N-x-S/T, where x is any amino acid except P. Notably, monocot and dicot PGIPs clearly form separate clusters (Fig 2), and CkPGIP1 clusters with dicotyledons such as *Solanum* and fruits such as *Actinidia deliciosa* and *Vaccinium corymbosum*.

### *CkPGIP1* transcripts accumulate in response to various stimuli

**Fig 3:**
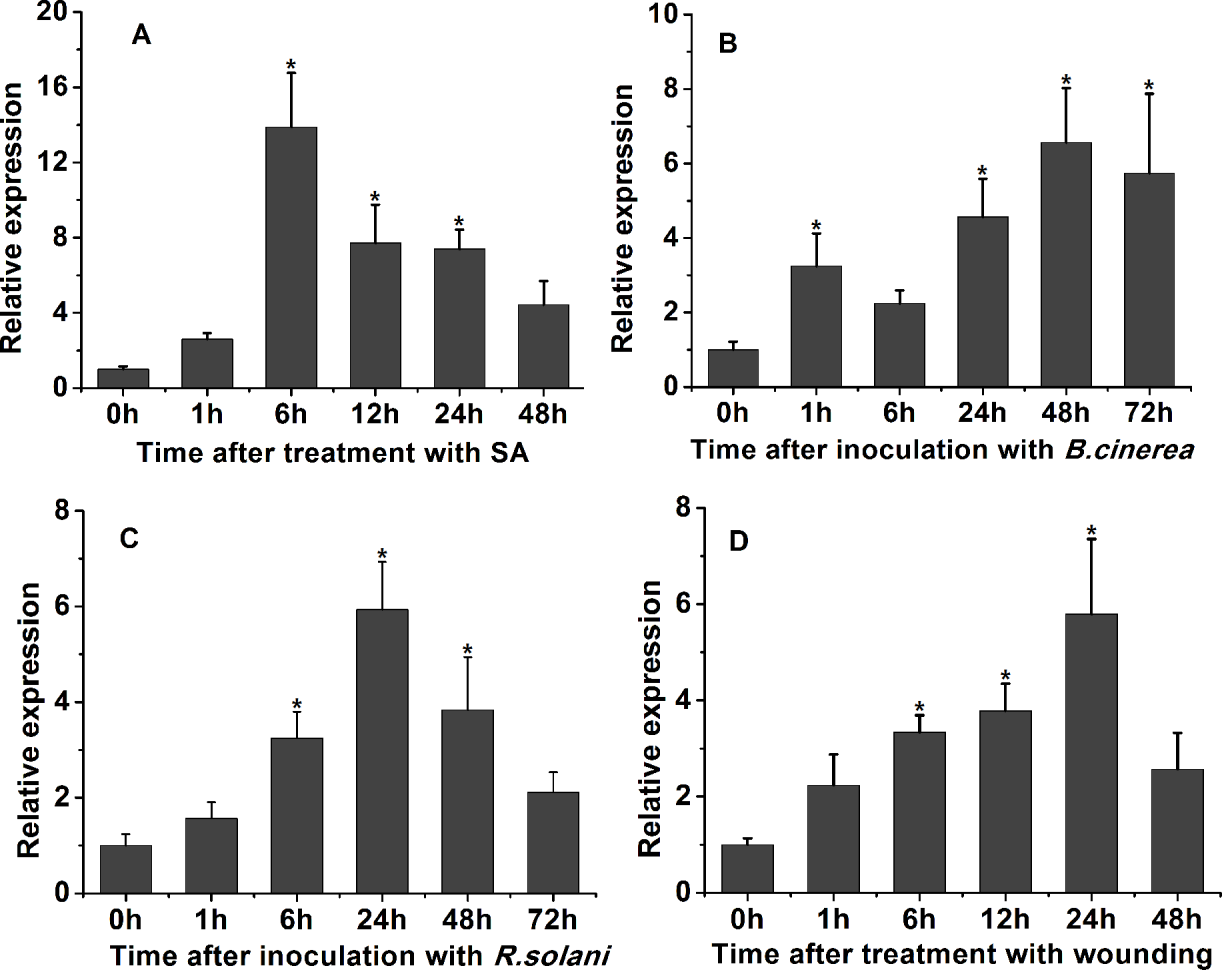
Analysis of *CkPGIP1* expression by qPCR. **A.** *CkPGIP1* expression 0, 1, 6, 12, 24 and 48 h after induction with salicylic acid. **B-C.** *CkPGIP1* expression 0, 1, 6, 24, 48 and 72 h after inoculation with *Botrytis cinerea* (**B**) and *Rhizoctonia solani* (**C**). **D.** *CkPGIP1* expression 0, 1, 6, 12, 24 and 48 h after wounding. Expression was compared to control plants, which were treated with double distilled water. Data were collected from three independent biological replicates. The data are means± standard errors (n = 3). Asterisks indicate a significant difference compared with control [least significance differences (LSD), *P < 0.05].

*CkPGIP1* expression was measured in response to salicylic acid, wounding, and fungal infection in *C*. *komarovii*. Salicylic acid [32, 33] induced expression 13.709 ± 2.041-fold over the basal level 6 h after exposure, at which expression remained stable before decreasing to 4.109 ± 0.672-fold at 48 h (Fig 3A). Infection with *B*. *cinerea* also increased expression 3.438 ± 0.612-fold at 1 h, before peaking to 6.432 ± 0.953-fold at 48 h and then diminishing to 5.551 ± 1.304-fold at 72 h (Fig 3B). *R*. *solani* infection elicited a similar response, and expression climbed to 3.145 ± 0.434-fold at 6 h, before peaking at 24 h to 5.874 ± 0.861-fold, and declining to 2.454 ± 0.857-fold three days after infection (Fig 3C). Finally, wounding gradually increased expression of *CkPGIP1* to 3.747 ± 0.579-fold at 12 h, and to 5.790 ± 1.567-fold at 24 h. Expression decreased thereafter (Fig 3D). Taken together, the data indicate that *CkPGIP1* expression is responsive to various stimuli.

### Homology modeling

Homology models of CkPGIP1 (S2A Fig), and polygalacturonase from *B*. *cinerea* (S2B Fig) and *R*. *solani* (S2C Fig) were generated based on X-ray crystal structures of PvPGIP2 (1OGQ) and of enzymes from *Colletotrichum lupini* (2IQ7) and *Chondrostereum purpureum* (1KCD). Geometry was optimized using KoBa^MIN^ (Rodrigues), and final models were found by MolProbity to have ideal geometry. Models were subsequently used in docking experiments.

### Docking studies

**Fig 4:**
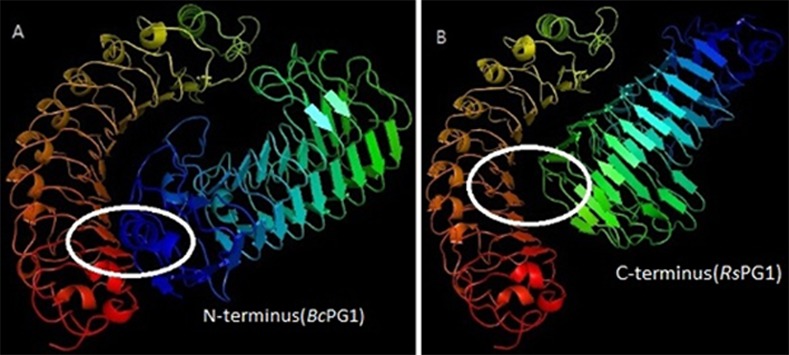
Docking studies of CkPGIP1 with polygalacturonase from *Botrytis cinerea* and *Rhizoctonia solani*. **A-B.** A concave site in CkPGIP1 interacts with the B1-sheet at the N-terminus of *B*. *cinerea* polygalacturonase (**A**), and with the C-terminus of *R*. *solani* polygalacturonase (**B**). When in complex with CkPGIP1, the substrate-binding site in the *B*. *cinerea* enzyme appears less exposed than in the enzyme from *R*. *solani* (circled in white).

PatchDock (Schneidman-Duhovny) was used to predict complexes between CkPGIP1 and polygalacturonase. Of the 20 highest-scoring, energy-minimized predicted complexes, 10 were re-refined in FireDock, and rescored based on binding energy, softened attractive and repulsive van der Waals energy, atomic contact energy, hydrogen and disulfide bonds, and ligand transformation. Structural changes due to re-refinement and optimization are summarized in S1 Table, and the highest-scoring complexes are depicted in Fig 4A and 4B.

In PGIPs, the binding site for polygalacturonase is a solvent-exposed concave surface comprised of β-strand/β-turn motifs in the central LRR domain [34]. Notably, LRRs are universally used as protein recognition domains in ~14,000 proteins [35]. Our docking studies indicated that the C-terminus of *Rhizoctonia* polygalacturonase interacts with CkPGIP1 at this surface (Fig 4B), while the N-terminus of the *Botrytis* polygalacturonase binds the B1 sheet (Fig 4A) with stronger affinity. As a result, the substrate-binding site in *Botrytis* polygalacturonase appears to be less exposed than in *Rhizoctonia*. In addition, the enzymes are in different orientations when bound to the inhibitor, as has been observed [29].

### Analysis of protein-protein interactions

Protein Interaction Calculator was used to investigate protein-protein interactions. Plots of putative exposed amino acids (Table 1) clearly suggested that the enzyme and inhibitor interact through hydrophobic interactions, hydrogen-bonding and ionic forces between exposed residues. At the surface, the dominant modes of interaction are ionic and hydrophobic. This analysis also confirmed that CkPGIP1 binds *Botrytis* polygalacturonase with stronger affinity than *Rhizoctonia* polygalacturonase.

**Table 1:** Interacting residues between CkPGIP1 and polygalacturonase from *Botrytis cinerea* and *Rhizoctonia solani*, as detected by Protein Interaction Calculator.

Interacting residues between		Interacting residues between	
CkPGIP1	BcPGI	CkPGIP1	BcPGI
*Hydrophobic interactions*			
I54	Y81	V71	Y115
M104	F128	W109	P133
A118	P149	L147	K150
P176	F217		
V240	P299		
Y277	A51		
L309	P308		
*Side chain-side chain hydrogen bonds between exposed residues*			
T79	S104	S60	S42
K124	Q151	T161	Q184
T161	D183	M232	R200
S214	R214		
N274	D161		
N301	H70		
*Ionic interactions between exposed residues*			
K44	E82	D73	D54
L74	K105	K159	K140
D135	K177	R206	E177
R157	D204		
H182	D182		
L216	E247		
R302	K239		

### Inhibitory activity of purified recombinant CkPGIP1

**Fig 5:**
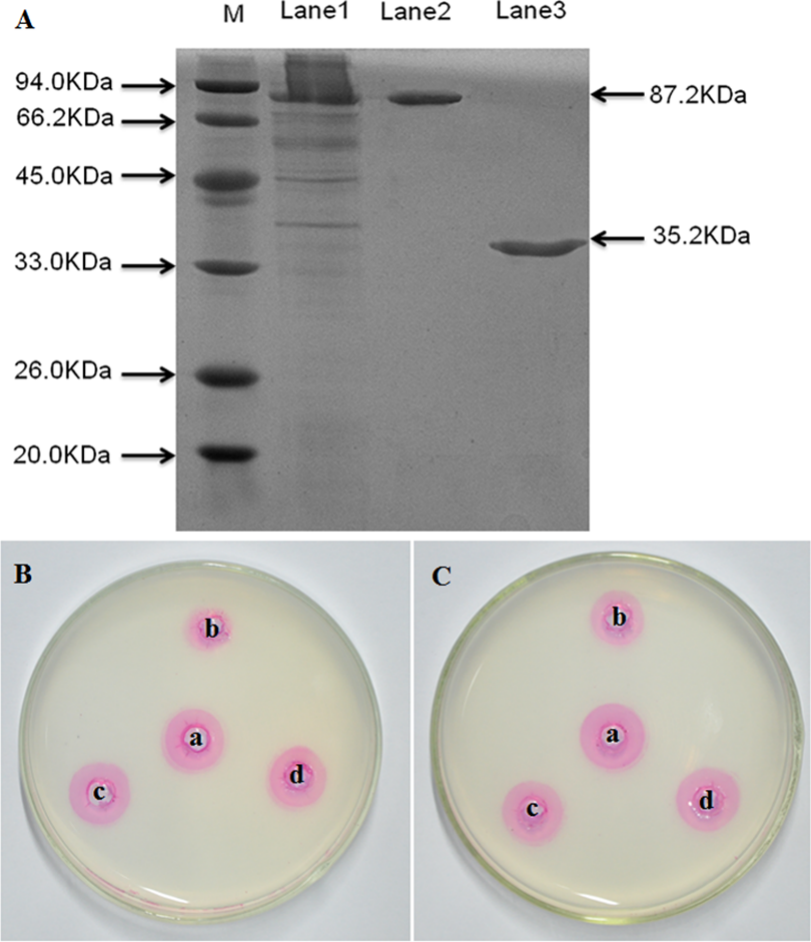
Purification and characterization of recombinant CkPGIP1. **A.** Lane 1, total protein extract from *E*. *coli* expressing recombinant CkPGIP1; Lane 2, flow through from a Ni-IDA superflow column; Lane 3, purified CkPGIP1, with fusion tags removed. **B-C.** Agarose diffusion assay of *Botrytis cinerea* (**B**) and *Rhizoctonia solani* polygalacturonase (**C**) in the presence or absence of purified recombinant CkPGIP1. a, 25 μL enzyme; b, 25 μL enzyme + 15 μg CkPGIP1; c, 25 μL enzyme + 25 μL phosphate-buffered saline; d, 25 μL enzyme + 15 μg heat-denatured CkPGIP1. Inhibitory activity is inversely proportional to the size of the ring, and the present of inhibition is indicated by a smaller ring size between comparisons.

CkPGIP1 was cloned in an expression vector designed for low-temperature expression, expressed in *Escherichia coli* BL21 (DE3) (S3 Fig), and purified according to published methods [36, 37], with some modification. And the protein migrated on SDS-PAGE with molecular weight 35.2 kDa (Fig 5A). Inhibitory activity of CKPGIP1 was measured by agarose diffusion assay [38]. Results indicated that CkPGIP1 inhibits *Botrytis* and *Rhizoctonia* polygalacturonase (Fig 5B and 5C) with IC_50_ 52.19 μg/mL and 64.13 μg/mL, respectively (Table 2).

**Table 2:** CkPGIP1 IC_50_ against fungal pathogens. Eight concentrations of purified CkPGIP1 were measured. Buffer was used as control.

Pathogen	IC_50_(μg/mL)	
*Botrytis cinerea*		52.19
*Rhizoctonia solani*		64.13
*Verticillium dahliae*		145.56
*Fusarium oxysporum*		206.71
*Valsa mali*		221.59

PGIP uses competitive, non-competitive, and mixed modes of inhibition depending on the target polygalacturonase, indicating that the inhibitor recognizes different structural motifs [39]. Indeed, PGIPs from different sources may inhibit the same polygalacturonase by different modes. For example, pear and bean PGIP inhibit *B*. *cinerea* polygalacturonase through competitive and mixed-mode mechanisms, respectively [39, 40].

### Subcellular localization

**Fig 6:**
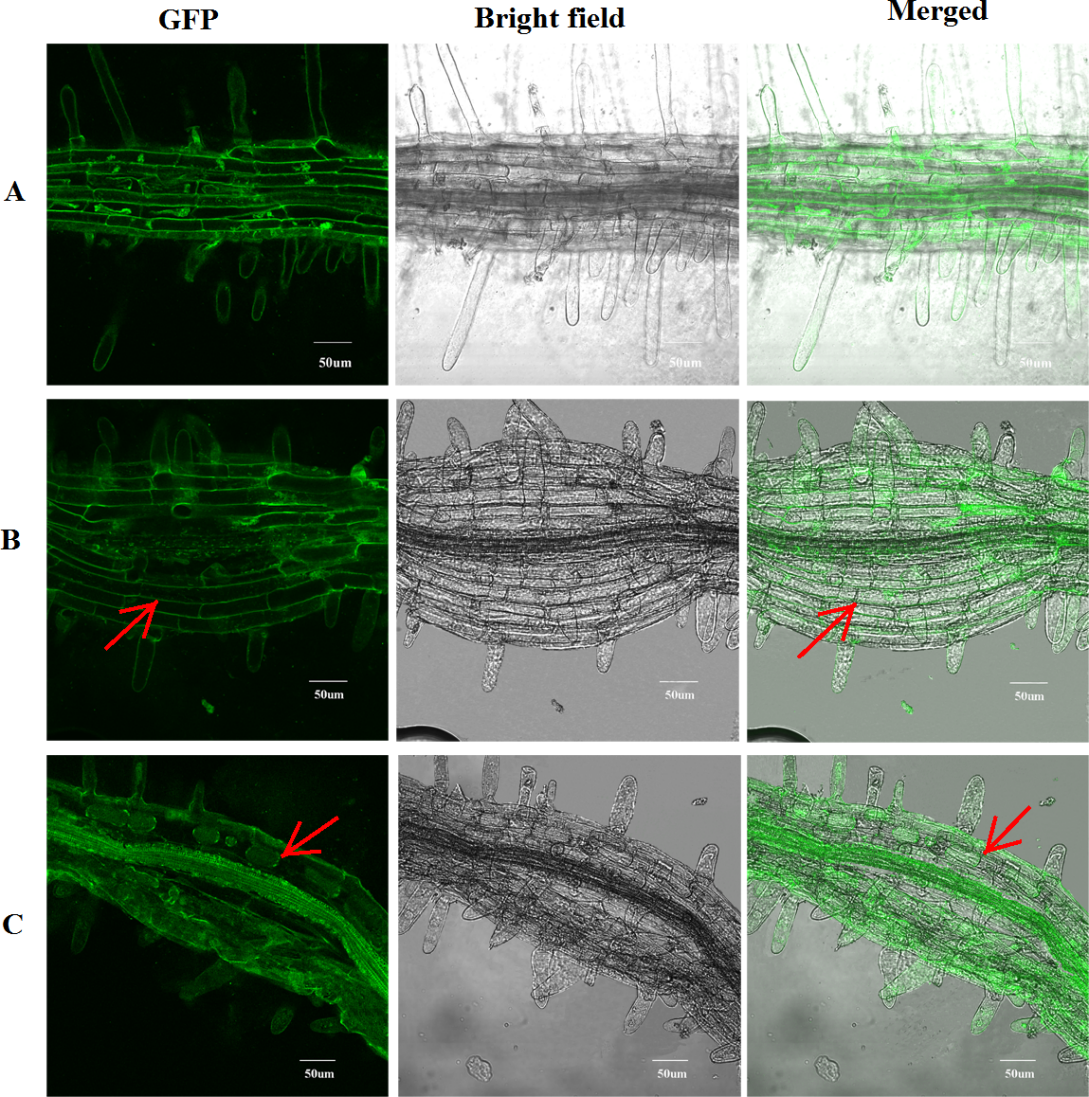
Subcellular localization of CkPGIP1::GFP and Super1300::GFP in transgenic Arabidopsis. **A.** Super1300::GFP. **B.** Normal CkPGIP1::GFP. **C.** plasmolyzed CkPGIP1::GFP. Plasmolysis was induced with 0.8 M mannitol for 10 min.

Subcellular localization was experimentally determined using a Super1300::CkPGIP1-GFP reporter fusion (S4 Fig). Then, the subcellular location of the CkPGIP1 within plant cell was assessed by the fusion protein CkPGIP1::GFP. Bright fluorescence was observed in extracellular space of seedling root cells by Confocal Laser Scanning Microscopy (Fig 6). The result showed that CkPGIP1 was located neither in the cytoplasm nor in the nucleus. And the red arrow in Fig 6B indicated that CkPGIP1 was located in the cell wall or in the plasma-membrane. Which was consistent with the predicted that CkPGIP1 would be located in the extracellular space (ProComp version 9.0 program). In order to differentiate between the plasma membrane and cell wall location, the seedlings were treated with 0.8M mannitol for 10 min. The arrow in Fig 6C indicated that the fluorescence was in the plasma membrane after plasmolysis (Fig 6C), suggesting that CkPGIP1 was localized to the plasma membrane.

### Transgenic overexpression in Arabidopsis

To identify transgenic Arabidopsis that stably expresses *CkPGIP1*, 14 plants were tested by PCR after screening for acquired tolerance to hygromycin. Of these, 11 were verified to be transgenic, and real-time PCR suggested that *CkPGIP1* is expressed to varying degrees (S5 Fig), with highest expression in line 9. Lines 14 and 3 expressed the transgene at slightly lower levels, and at significantly lower levels in all other lines. Therefore, lines 9 and 14 were selected for further experiments.

### Fungal challenge in transgenic Arabidopsis

**Fig 7:**
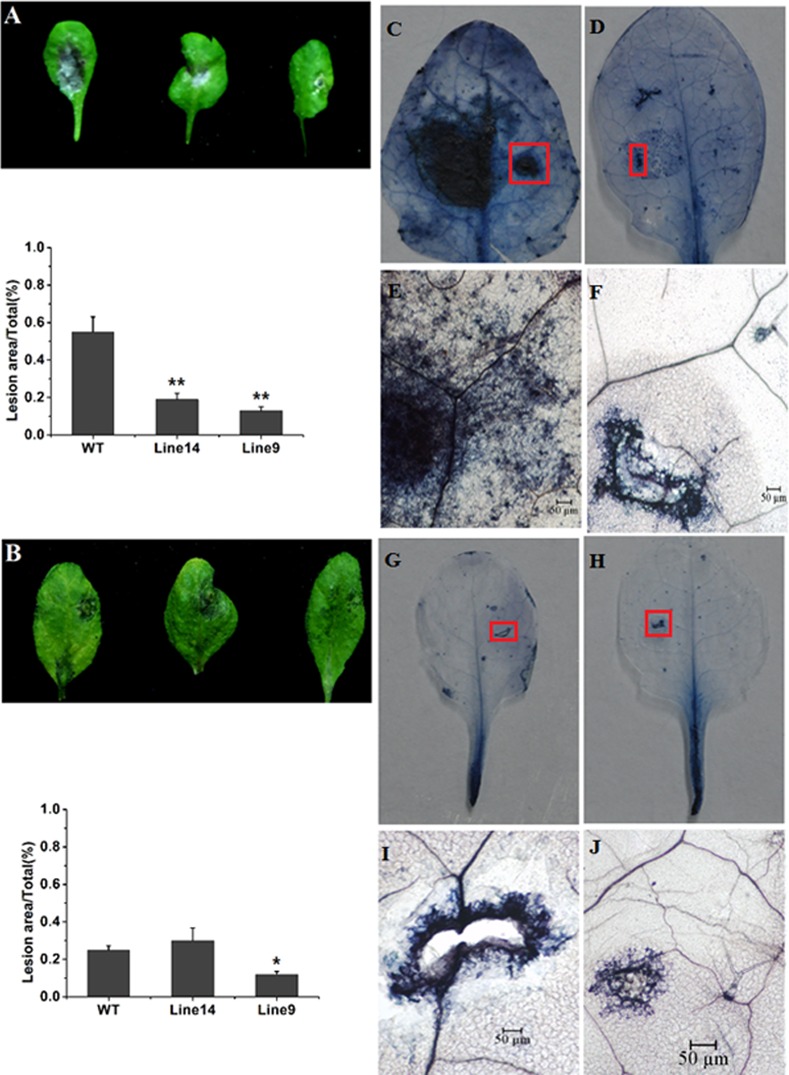
Transgenic expression of CkPGIP1 increases resistance to *Botrytis cinerea* and *Rhizoctonia solani* in Arabidopsis. **A-B.** Disease symptoms (top panel) and lesions (bottom panel) 6 d after wild type and transgenic Arabidopsis were inoculated with *B*. *cinerea* (**A**), and 5 d after inoculation with *R*. *solani* (**B**). Error bars are standard error (n = 3). *, *P* < 0.05 and **, *P* < 0.01 by least significant difference against wild type. **C**-**D.** Trypan blue staining of wild type **(C)** and transgenic Arabidopsis leaves **(D)** 4 d after inoculation with *B*. *cinerea*. **E-F.** Zoomed-in view of disease symptoms boxed in red in **(C)** and **(D)**. **G**-**J.** Trypan blue staining of wild-type **(G)** and transgenic Arabidopsis leaves **(H)** at 3 d post-inoculation with *R*. *solani*. (**H**) and (**I**) are enlarged views of areas boxed in red in (**G**) and (**H**).

Wild type and transgenic Arabidopsis were drop-inoculated with a suspension of *B*. *cinerea* conidia as described previously [41]. Symptoms were obvious six days after inoculation, and lesions were visibly smaller in transgenic plants (Fig 7A). Notably, symptoms in transgenic Arabidopsis were restricted to the inoculation site, particularly in line 9. In addition, lesions due to *R*. *solani* five days post-infection were smaller in line 9 than in wild type plants, although the largest lesions were observed in line 14. However, etiolation in veins and leaves was clear in wild type plants, but not in transgenic lines (Fig 7B).

Furthermore, infected leaves were stained with trypan blue to assess fungal growth. Results indicated that *B*. *cinerea* germination and growth were significantly restricted in transgenic plants than in wild type plants, suggesting enhanced resistance (Fig 7C–7F). However, growth of *R*. *solani* was not visibly different between wild type and transgenic lines (Fig 7G–7J).

### Inhibitory activity of Arabidopsis-expressed CkPGIP1

**Fig 8:**
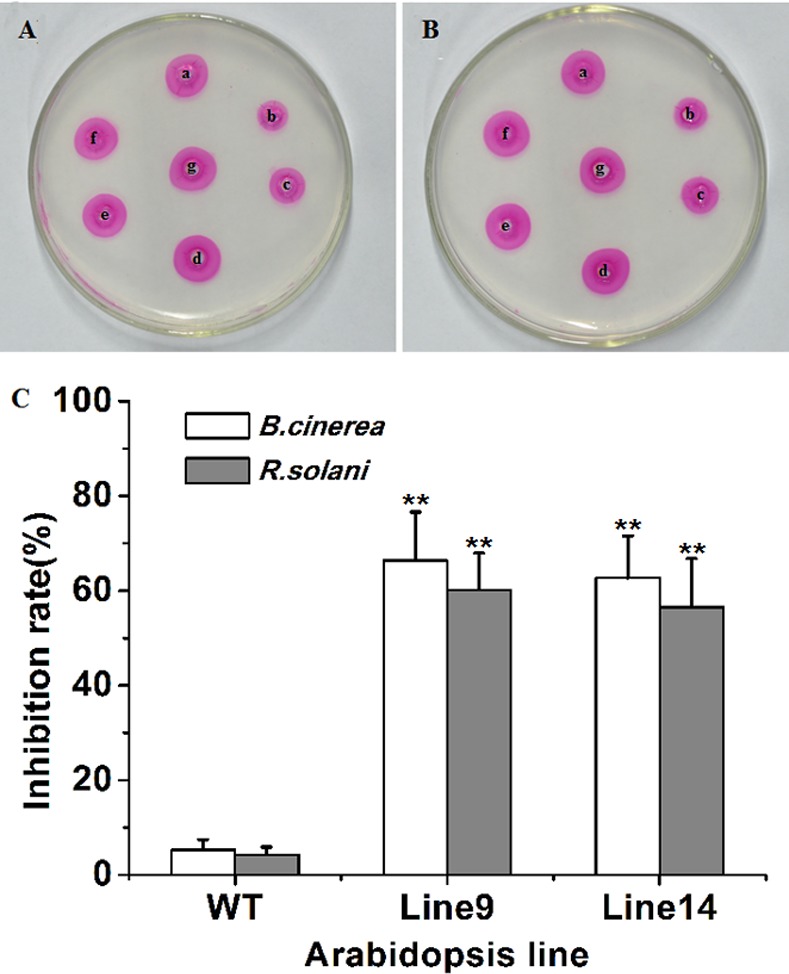
Inhibitory activity of crude protein extracts from wild type and transgenic Arabidopsis. Inhibitory activity was measured against *Botrytis cinerea* (**A**) and *Rhizoctonia solani* polygalacturonase (**B**). a-c, 15 μL crude enzyme + 15 μg crude protein extract from wild type and transgenic Arabidopsis lines 9 and 14; d-f, 15 μL crude enzyme + 15 μg heat-denatured crude protein extract from wild type and transgenic Arabidopsis lines 9 and 14; g, crude PGs. **C.** Inhibition rate of crude protein extracts from wild type and transgenic Arabidopsis. Error bars indicate standard error (n = 3). **, *P* < 0.01 by least significant difference against wild type.

Agarose diffusion assays suggested that crude protein extracts from transgenic Arabidopsis inhibit fungal polygalacturonase more effectively than extracts from wild type plants (Fig 8A and 8B). Indeed, crude CkPGIP1 inhibited *Botrytis* and *Rhizoctonia* polygalacturonase by 62.7–66.4% and 56.5–60.2%, respectively (Fig 8C). All inhibitory activity was lost when crude CkPGIP1 was denatured by boiling.

## Discussion

Polygalacturonase-inhibiting protein, a LRR protein organized into multigene families in plants [42], is a key agent of pathogen-associated molecular pattern-triggered immunity in flowering plants [43]. In this study, we characterized CkPGIP1 from *C*. *komarovii*. The inhibitor is phylogenetically similar to homologs in *Solanum*, *A*. *deliciosa*, and *V*. *corymbosum* (Fig 2), and is predicted to be N-glycosylated at five sites within β-strand/β-turn regions. Notably, N-glycosylation is critical for ligand binding and disease resistance [44], as demonstrated in PGIPs from *Pennisetum glaucum* and *P*. *vulgaris*, which have seven and three N-glycosylation sites, respectively, and which effectively inhibit polygalacturonase from *A*. *niger* and *F*. *moniliforme* [45].

Bioinformatic analysis and subcellular localization experiments showed that CkPGIP1, is a non-transmembrane protein targeted to the cell wall or plasma membrane (Fig 6B). Then further plasmolysis experiment was taken, and results showed that CkPGIP1 was located in the plasma membrane (Fig 6C), which was consistant with the previous study, such as PGIP from *Oryza sativa* [46].

Like other PGIPs and PGIP-like proteins, CkPGIP1 contains a number of conserved LRR units (Fig 1), which have been reported to trigger defense response either by interacting with other proteins [2, 47]. Subtle differences in the structure of LRR domains, in addition to glycosylation of specific residues, contribute to the variability of inhibitory activities against different polygalacturonases [48, 49].

Furthermore, structural differences may also confer specificity, as has been observed in *P*. *vulgaris*, the PGIPs of which recognize and inhibit several enzymes to varying extent [13, 23, 50]. The enzymes themselves likely contribute to specificity by presenting diverse surfaces with different electrostatic potential. Accordingly, our docking studies indicated that CkPGIP1 uses different sites to bind the N-terminus and C-terminus of *Botrytis* and *Rhizoctonia* polygalacturonase, respectively. As a result, some residues are critical to bind one target enzyme, but are dispensable to bind another, suggesting that different but overlapping subsets of residues are functionally important [50].

In particular, the complex between CkPGIP1 and *Botrytis* polygalacturonase is formed via a larger number of residues. Consequently, CkPGIP1 binds this enzyme with stronger affinity than *R*. *solani* polygalacturonase. Notably, the active site of *Botrytis* polygalacturonase is partially buried when in complex with CkPGIP1, and the substrate-binding site appears shielded, in line with the typical mode of inhibition [28, 39]. Collectively, these observations indicate that PGIP interacts with target enzymes using different structural elements, and binding modes.

CkPGIP1 inhibits polygalacturonases from a variety of pathogenic fungi, but is most effective against enzymes from *B*. *cinerea* and *R*. *solani* (Table 2). Accordingly, transgenic expression of *CkPGIP1* in Arabidopsis significantly limits the spread and growth of fungal infections. In particular, trypan blue staining clearly showed that *B*. *cinerea* virulence and growth is significantly limited in transgenic Arabidopsis, indicating that CkPGIP1 directly determines the ability of fungal pathogens to degrade plant tissues. However, overexpression of *CkPGIP1* in transgenic Arabidopsis didn’t exhibit evident resistance to *R*. *solani*, as lesion size and trypan blue staining were not obviously different between wild type and transgenic Arabidopsis (Fig 7). In any case, crude protein extracts from transgenic Arabidopsis specifically inhibits 62.7–66.4% and 56.5–60.2% of *B*. *cinerea* and *R*. *solani* polygalacturonase activity (Fig 8A–8C).

PGIP expression is regulated through different transduction pathways [33, 51], perhaps to precisely calibrate the response to a wide range of biotic and environmental cues, including pathogen infection [52–54]. It is also clear that plants may have evolved mechanisms that integrate various stress signals in order to mount a coordinated, coherent response [51, 55]. For instance, salicylic acid and wounding trigger the same response, as demonstrated in *C*. *annuum* [32] and other species [56–58]. Indeed, salicylic acid is a key signaling molecule in defense mechanisms, and accumulates in many species along with pathogenesis-related genes. Therefore, our data suggest that *CkPGIP1* not only triggers a sophisticated response to pathogen infection, but may also activate additional defense mechanisms that are typically triggered by environmental stimuli via salicylic acid signaling.

Altogether, our results demonstrate the antifungal nature of CkPGIP1 and its ability to enhance disease resistance in transgenic plants, as well as highlight its potential application in biotechnology. Further investigation of the interaction molecular mechanisms between polygalacturonase and PGIP is crucial in the design of pathogenic factors with host PGIP proteins and may uncover mechanisms of pathogen invasion. This study combining molecular docking simulation with *in vitro* fungicidal activities of CkPGIP1, identified plant defense molecules that are specifically targeted by multiform pathogens, as well as proteins that may otherwise be used to stimulate resistance.

## Materials and Methods

### Plant materials and preparation of fungal polygalacturonase

*C*. *komarovii* seedlings were cultivated in standard conditions at 25°C and 16-h photoperiod. Samples were collected after one month and stored at -80°C. Virulent strains of *B*. *cinerea*, *Verticillium dahliae*, *Fusarium oxysporum f*. *sp*. *tulipae*, *R*. *solani*, and *Valsa mali* were cultured on potato dextrose agar (200 g L^-1^ potato, 20 g L^-1^ dextrose, and 15 g L^-1^ agar) at 25°C for a week, when cells are most active [59]. Colonies were then inoculated into 500 mL Erlenmeyer flasks containing 100 mL Czapek’s liquid medium, and grown at 25°C and 200 rpm for 7 d. Cells were pelleted by centrifugation at 12,000 rpm at 4°C. Crude polygalacturonase was obtained by dialyzing the resulting supernatant at 4°C against 0.1 M sodium acetate-acetic acid buffer pH 4.8, and then concentrating by ultrafiltration, with molecular weight cutoff of 10 kD.

### Cloning

Total RNA was extracted from *C*. *komarovii* complete stool using a commercially available kit (Promega, WI, USA). Polyadenylated mRNA was obtained using PolyATract mRNA Isolation System (Promega, WI, USA). Subsequently, a cDNA library was generated using a cDNA Library Construction Kit (Merck, Germany). The library was propagated on 150 mm plates to obtain about 10^6^ plaques [30]. A conserved 21-amino acid sequence (5’-FDXSYFHNKCLCGAPLPSCK-3’) at the C-terminus of all previously characterized PGIPs [31] was used to probe the library by colony *in situ* hybridization. A positive plaque was obtained after three rounds, and the fragment was subcloned into pBlueScript II SK (+) through *in vivo* excision, following the manufacturer’s protocol.

### Sequence and phylogenetic analysis

The sequence encoding CkPGIP1 was determined by a homology search of NCBI databases. Phylogenetic analysis was carried out in MEGA 5.1. Clustal Omega and SMART were used for multiple sequence alignment and domain prediction, respectively.

### Subcellular localization

*CkPGIP1* was amplified using primers with sequence 5'-ggC gTC gAC ATg AAg AAg ATT TCT TCT CTg-3' and 5'-TAg TCT AgA CTT gCA AgA Agg CAA Agg A-3', and which incorporate *Sal*I and *Xba*I cleavage sites, respectively. Templates were initially denatured at 94°C for 3 min, and then amplified over 35 cycles at 94°C for 30 s, 45–55°C for 30 s, and 72°C for 1 min, with a final extension step at 72°C for 7 min. The amplified fragment was cloned into pMD18-T, sequenced, and subcloned into Super-pCAMBIA1300. The intracellular localization of CkPGIP1 was determined by CkPGIP1::GFP fusion protein in transgenic Arabidopsis. The roots of 7-day-old transgenic seedlings were detected by FLUOVIEW FV1000 Confocal Laser Scanning Microscopy (OLYMPUS, Tokyo, Japan). Excitation light at 488 and 543 nm was attenuated to 50% transmittance. Plasmolysis was induced by incubating samples in 0.8 M mannitol for 10 min.

### RNA isolation and real-time PCR

Total RNA was isolated using an RNA Extraction Kit (CW BIO) from the complete stool of *C*. *komarovii* plants that were wounded, exposed to 5 mM salicylic acid [32], or infected with *B*. *cinerea* and *R*. *solani* cultivated in PDA (Difco) plates [55]. First-strand cDNA was synthesized by Fast Quant cDNA Reverse Kit (TIANGEN BIOTECH CO., LTD), diluted, and used as template for quantitative real-time PCR. *CkPGIP1* was amplified using primers with sequence 5'-gAT ACC ggA TgC TgT ggg Tg-3' and 5'-ggA ggg ATT gAA CCg gTg Ag-3'. The endogenous gene *EF-1-ɑ* [30] was used as control. Reactions were prepared in 20 μL using SYBR®Premix Ex Taq (Tli RNaseH Plus) (Takara, Shiga, Japan), and amplified on an ABI 7500 thermocycler (Applied Biosystems, Foster city, CA, USA). Expression was determined by the 2^-ΔΔCT^ method. Data were analyzed in Origin 8 as described [30].

### Homology modeling

Initial homology models of CkPGIP1 and polygalacturonase from *B*. *cinerea* and *R*. *solani* were generated using SWISS-MODEL. *P*. *vulgaris* PvPGIP2 (1OGQ) was used as template for CkPGIP1, with which it shares 48% sequence identity. *B*. *cinerea* polygalacturonase was modeled based on endopolygalacturonase I from *C*. *lupini* (2IQ7), to which it is 57% identical. Finally, endopolygalacturonase I from *Chondrostereum purpureum* (1KCD) was selected as template for *R*. *solani* polygalacturonase on the basis of 68% sequence identity. Models were refined in KoBa^MIN^ server (Rodrigues), and visualized in Pymol.

### Docking and energy minimization

To obtain relatively precise ensembles of complexes between CkPGIP1 and polygalacturonases, a search for possible complexes was first performed in PatchDock (Schneidman-Duhovny). Search results were then re-refined and minimized in FiberDock.

### Analysis of interaction surfaces

Protein Interaction Calculator was used to investigate interaction forces between CkPGIP1 and polygalacturonases. In particular, we examined hydrophobic interactions, side chain-side chain hydrogen bonds, and intramolecular ionic interactions between exposed residues, the interactions were visualized and analyzed using molecular modeling programs PyMOL 1.6 and CHIMERA 1.8 [60].

### Expression and purification of CkPGIP1

CkPGIP1 was amplified using primers 5'-CgC ggA TCC gCC ACT AAA AAA gAA AAg TgC-3' and 5'-TCg CTC gAg TTA TTA TTA CTT gCA AgA Agg CAA Agg A-3', and cloned into pCold TF DNA, a vector designed for low-temperature expression in bacteria. The construct was transformed into *E*. *coli* BL21 (DE3), and single colonies were cultured at 37°C in Luria-Bertani broth supplemented with 100 μg/mL ampicillin. To scale up, pre-cultures were inoculated at 1% into fresh media. At OD 0.4~0.5, cultures were placed for 30 min without shaking at 15°C, immediately induced with 1.0 mM IPTG, and cultured for another 24 h at 15°C with oscillation. Cells were harvested by centrifugation for 20 min at 10,000 *×g*. Soluble expression was verified by SDS-PAGE, and CkPGIP1 was purified using 6× His-Tagged Protein Purification Kit (CW BIO). Subsequently, the fusion tags 6× His and trigger factor, which are encoded in the vector, were removed by Thrombin Cleavage Capture Kit (Novagen).

### Inhibitory activity assay

Inhibitory activity was measured in agarose plate diffusion experiments using 0.8% agarose and 0.5% PCA in 0.1 M sodium acetate-acetic acid buffer pH 4.8. Polygalacturonases were spotted with or without CkPGIP1 on agarose plates, and incubated at 30°C for 12 h. Thereafter, the gel was stained with 0.05% w/v ruthenium red in water, and then thoroughly rinsed with sterile water [38]. Enzymatic activity was measured in agarose diffusion units, which represent the amount of enzyme required to produce a ring with radius 0.5 cm. The size of the ring is inversely proportional to inhibitory activity.

In addition, polygalacturonase activity was also measured in reducing units. A reducing unit is the amount of enzyme required to release reducing groups from d-galacturonic acid at 1 mol min^-1^. Inhibitory activity was measured against 0.0011 reducing units, and the amount of CkPGIP1 required to reduce hydrolysis by 50% was defined as 1 unit activity [32].

### Fungal challenge in transgenic Arabidopsis

*B*. *cinerea* conidia were prepared as described [61], while *R*. *solani* was initially propagated on potato dextrose agar at 25°C. Soil-grown plants were challenged with *B*. *cinerea* by drop-inoculation [41]. On the other hand, wild type and transgenic Arabidopsis were challenged with *R*. *solani* at 5 weeks by *in vitro* back leaf inoculation. Briefly, blades were sheared, and placed with back up on filter paper in a 9 cm Petri dish containing sterile water. Bacterial debris was removed by pressure, and 3 μL *R*. *solani* filtrate was spotted at four sites per leaf. Infected leaves were then incubated in the dark at 25°C.

To measure fungal growth, infected leaves were stained with trypan blue. A stock solution of trypan blue was prepared with 10 mL 85% lactic acid, 10 mL phenol, 10 mL distilled water, and 10 mg trypan blue (Sigma-Aldrich). A working solution was then prepared by diluting the stock 1:1 in 95% ethanol. Infected leaves were incubated in working solution, boiled for 1 min, cooled, and left at room temperature overnight. Chloral hydrate (1.25 mg/mL) was then used to remove chlorophyll [62], and leaves were examined and photographed under a 4×/0.25 numerical aperture objective in a Nikon eclipse Ti microscope (Japan).

### Extraction and assay of crude CkPGIP1 from transgenic Arabidopsis

Crude protein extracts containing PGIPs were prepared from leaves of wild type and T3 transgenic Arabidopsis lines 9 and 14 after infection with both fungi [32]. The inhibitory activity of crude CkPGIP1 from transgenic Arabidopsis was analyzed as described for *E*. *coli*-expressed proteins.

## Supporting Information

S1 Fig cDNA and amino acid sequence of *CkPGIP1* from *Cynanchum komarovii*. The signal peptide and N-glycosylation sites are highlighted in gray and yellow, respectively. LRRNT-2 and LRR domains are singly and doubly underlined, respectively. Cysteines are marked with diamonds.
(TIF)

S2 Fig Homology modeling. Homology models of CkPGIP1 (**A**), *Botrytis cinerea* (**B**), and *Rhizoctonia solani* polygalacturonase (**C**) are based on known structures of PvPGIP (1OGQ), and polygalacturonase from *C*. *lupini* (2IQ7), and *Chondrostereum purpureum* (1KCD).
(TIF)

S3 Fig Schematic representation of pCold-TF-CkPGIP1 bacterial expression plasmid. (TIF)

S4 Fig Schematic representation of the plant expression vector pCAMBIA1300-CkPGIP1. (TIF)

S5 Fig Selection and identification of transgenic Arabidopsis. Lines 1–14 were genotyped by PCR, and 11 were found to be transgenic. Real-time PCR was then used to measure relative expression of *CkPGIP1*.
(TIF)

S1 Table The comparison of all the changes about the complexes before and after optimization. (**A**): before optimization;(**B**): after optimization. glob: the binding energy of the solution; aVdWa and rVdW: softened attractive and repulsive van der Waals energy; ACE: atomic contact energy; HB: hydrogen and disulfide bonds.
(DOCX)

## References

[1] Bézier A, Lambert B, Baillieul F (2002) Study of defense-related gene expression in grapevine leaves and berries infected with *Botrytis cinerea*. Eur. J. Plant Pathol 108: 111–120.

[2] Jones DA, Jones JDG (1997) The role of leucine-rich repeat proteins in plant defences. Adv. Bot. Res 24:90–168.

[3] Isshiki A, Akimitsu K, Yamamoto M, Yamamoto H (2001) Endopolygalacturonase is essential for citrus black rot caused by *Alternaria citri* but not brown spot caused by *Alternaria alternata*. Mol. Plant-Microbe Interact 14:749–757. 11386370 10.1094/MPMI.2001.14.6.749

[4] Karr AL, Albersheim P (1970) Polysaccharide-degrading enzymes are unable to attack plant cell walls without prior action by a “wall-modifying enzyme”. Plant Physiol 46:69–80. 16657425 10.1104/pp.46.1.69 PMC396536

[5] Have AT, Mulder W, Visser J, van Kan JA (1998) The endopolygalacturonase gene *Bcpg1* is required for full virulence of *Botrytis cinerea*. Mol. Plant-Microbe Interac 11:1009–1016. 10.1094/MPMI.1998.11.10.1009 9768518

[6] Kars I, Krooshof GH, Wagemakers L, Joosten R, Benen JA, Van Kan JA (2005) Necrotizing activity of five *Botrytis cinerea* endopolygalacturonases produced in Pichia pastoris. Plant J 43:213–225. 15998308 10.1111/j.1365-313X.2005.02436.x

[7] Shieh MT, Brown RL, Whitehead MP, Cary JW, Cotty PJ, Cleveland TE, et al (1997) Molecular genetic evidence for the involvement of a specific polygalacturonase, P2c, in the invasion and spread of *Aspergillus flavus* in cotton bolls. Appl Environ Microbiol 63: 3548–3552. 9293005 10.1128/aem.63.9.3548-3552.1997 PMC168660

[8] Oeser B, Heidrich PM, Müller U, Tudzynski P, Tenberge KB (2002) Polygalacturonase is a pathogenicity factor in the *Claviceps purpurea/rye* interaction. Fungal. Genet. Biol 36:176–186. 12135573 10.1016/s1087-1845(02)00020-8

[9] Centis S, Guillas I, Séjalon N, Esquerré-Tugayé MT, Dumas B (1997) Endopolygalacturonase genes from *Colletotrichum lindemuthianum*: cloning of *CLPG2* and comparison of its expression to that of *CLPG1* during saprophytic and parasitic growth of the fungus. Mol. Plant-Microbe Interac 10: 769–775. 10.1094/MPMI.1997.10.6.769 9245838

[10] Pietro AD, Roncero MIG (1998) Cloning, expression, and role in pathogenicity og pg1 encoding the major extracellular endopolygalacturonase of the vascular wilt pathogen Fusarium oxysporum. Mol. Plant-Microbe Interac. 11:91–98. 10.1094/MPMI.1998.11.2.91 9450333

[11] Sun WX, Jia YJ, Feng BZ, O'Neill NR, Zhu XP, Xie BY, et al (2009) Functional analysis of *Pcipg2* from the straminopilous plant pathogen *Phytophthora capsici*. Genesis 47:535 10.1002/dvg.20530 19422018

[12] Akagi A, Engelberth J, Stotz HU (2010) Interaction between polygalacturonase—inhibiting protein and jasmonic acid during defense activation in tomato against *Botrytis cinerea*. Eur J Plant Pathol 128:423–428.

[13] D’ovidio R, Roberti S, Di Giovanni M, Capodicasa C, Melaragni M, Sella L, et al (2006) The characterization of the soybean polygalacturonase-inhibiting proteins (Pgip) gene family reveals that a single member is responsible for the activity detected in soybean tissues. Planta 224: 633–645. 16501991 10.1007/s00425-006-0235-y

[14] Matteo Komjanc, Festi S, Rizzotti L, Cattivelli L, Cervone F, De Lorenzo G (1999) A leucine-rich repeat receptor-like protein kinase (LRPKm1) gene is induced in *Malus domestica* by *Venturia inaequalis* infection and salicylic acid treatment. Plant Mol. Biol 40:945–957. 10527419 10.1023/a:1006275924882

[15] Bennett A, Labavitch JM, Powell A, Stotz H (1996) U.S. Patent No. 5,569,830. Washington, DC: U.S. Patent and Trademark Office.

[16] Ferrari S, Vairo D, Ausubel FM, Cervone F, De Lorenzo G (2003) Tandemly duplicated Arabidopsis genes that encode polygalacturonase-inhibiting proteins are regulated coordinately by different signal transduction pathways in response to fungal infection. Plant Cell 15: 93–106. 12509524 10.1105/tpc.005165 PMC143454

[17] Joubert DA, Slaughter AR, Kemp G, Becker JV, Krooshof GH, Bergmann C, et al (2006) The grapevine polygalacturonase-inhibiting protein (VvPGIP1) reduces *Botrytis cinerea* susceptibility in transgenic tobacco and differentially inhibits fungal polygalacturonases. Transgenic. Res 15:687–702. 17072564 10.1007/s11248-006-9019-1

[18] Powell AL, van Kan J, ten Have A, Visser J, Greve LC, Bennett AB, et al (2000) Transgenic expression of pear PGIP in tomato limits fungal colonization. Mol. Plant-Microbe Interac 13:942–950. 10.1094/MPMI.2000.13.9.942 10975651

[19] Agueero CB, Uratsu SL, Greve C, Powell AL, Labavitch JM, Meredith CP, et al (2005) Evaluation of tolerance to Pierce's disease and Botrytis in transgenic plants of *Vitis vinifera* L. expressing the pear PGIP gene. Mol. Plant. Pathol 6:43–51. 10.1111/j.1364-3703.2004.00262.x 20565637

[20] Ferrari S, Galletti R, Vairo D, Cervone F, De Lorenzo G (2006) Antisense expression of the *Arabidopsis thaliana* AtPGIP1 gene reduces polygalacturonase-inhibiting protein accumulation and enhances susceptibility to *Botrytis cinerea*. Mol. Plant-Microbe Interac 19:931–936. 10.1094/MPMI-19-0931 16903359

[21] Desiderio A, Aracri B, Leckie F, Mattei B, Salvi G, Tigelaar H, et al (1997) Polygalacturonase-inhibiting proteins (PGIPs) with different specificities are expressed in *Phaseolus vulgaris*. Mol. Plant-Microbe Interac 10: 852–860. 10.1094/MPMI.1997.10.7.852 9304859

[22] Manfredini C, Sicilia F, Ferrari S, Pontiggia D, Salvi G, Caprari C, et al (2005) Polygalacturonase-inhibiting protein 2 of *Phaseolus vulgaris* inhibits *BcPG1*, a polygalacturonase of *Botrytis cinerea* important for pathogenicity, and protects transgenic plants from infection. Physiol. Mol. Plant Pathol 67:108–115.

[23] Mariotti L, Casasoli M, Migheli Q, Balmas V, Caprari C, De Lorenzo G (2008) WITHDRAWN: Reclassification of *Fusarium verticillioides* (*syn*. *F*. *moniliforme*) strain FC-10 as F. phyllophilum.Mycol. Res10.1016/j.mycres.2008.07.004 18672060

[24] Frediani M, Cremonini R, Salvi G, Caprari C, Desiderio A, D'ovidio R, et al (1993) Cytological localization of thePGIP genes in the embryo suspensor cells of *Phaseolus vulgavis L*. Theor. Appl. Genet 87: 369–373. 10.1007/BF01184925 24190264

[25] Misas-Villamil JC, Van der Hoorn RA (2008) Enzyme–inhibitor interactions at the plant–pathogen interface. Curr. Opin. Plant Biol 11:380–388. 10.1016/j.pbi.2008.04.007 18550418

[26] Di Matteo A, Federici L, Mattei B, Salvi G, Johnson KA, Savino C, et al (2003) The crystal structure of polygalacturonase-inhibiting protein (PGIP), a leucine-rich repeat protein involved in plant defense. Proc. Natl Acad. Sci 100:10124–10128. 12904578 10.1073/pnas.1733690100 PMC187787

[27] Prabhu SA, Singh R, Kolkenbrock S, Sujeeth N, Gueddari NEE, Moerschbacher BM, et al (2014) Experimental and bioinformatic characterization of recombinant polygalacturonase-inhibitor protein from pearl millet and its interaction with fungal polygalacturonases. Journal of Experimental Botany. 65: 5033–5047. 10.1093/jxb/eru266 24980909 PMC4144779

[28] Lim JM, Aoki K, Angel P, Garrison D, King D, Tiemeyer M, et al (2009) Mapping Glycans onto Specific N-Linked Glycosylation Sites of *Pyrus communis* PGIP Redefines the Interface for EPG− PGIP Interactions. J. Proteome Res 8:673–680. 10.1021/pr800855f 19072240 PMC4141487

[29] Maulik A, Ghosh H, Basu S (2009) Comparative study of protein-protein interaction observed in Polygalacturonase-inhibiting proteins from *Phaseolus vulgaris* and *Glycine max* and Polygalacturonase from Fusarium moniliforme. BMC genomics 10(Suppl 3), S19 10.1186/1471-2164-10-S3-S19 19958482 PMC2788371

[30] Wang Q, Li F, Zhang X, Zhang Y, Hou Y, Zhang S, et al (2011) Purification and characterization of a CkTLP protein from *Cynanchum komarovii* seeds that confers antifungal activity. PloS one 6, e16930 10.1371/journal.pone.0016930 21364945 PMC3043079

[31] De Lorenzo G, D'Ovidio R, Cervone F (2001) The role of polygalacturonase-inhibiting proteins (PGIPs) in defense against pathogenic fungi. Annu. Rev. Plant Physiol 39:313–335. 10.1146/annurev.phyto.39.1.313 11701868

[32] Wang X, Zhu X, Tooley P, Zhang X (2013) Cloning and functional analysis of three genes encoding polygalacturonase-inhibiting proteins from *Capsicum annuum* and transgenic CaPGIP1 in tobacco in relation to increased resistance to two fungal pathogens. Plant Mol. Biol 81:379–400. 10.1007/s11103-013-0007-6 23334855

[33] Li R, Rimmer R, Yu M, Sharpe AG, Séguin-Swartz G, Lydiate D, et al (2003) Two *Brassica napus* polygalacturonase inhibitory protein genes are expressed at different levels in response to biotic and abiotic stresses. Planta 217:299–308. 12783338 10.1007/s00425-003-0988-5

[34] Kobe B, Deisenhofer J (1994) The leucine-rich repeat: a versatile binding motif. Trends. Biochem. Sci 19:415–421. 7817399 10.1016/0968-0004(94)90090-6

[35] Matsushima N, Miyashita H (2012) Leucine-rich repeat (LRR) domains containing intervening motifs in plants. Biomolecules 2:288–311. 10.3390/biom2020288 24970139 PMC4030839

[36] Favaron F, D'ovidio R, Porceddu E, Alghisi P (1994) Purification and molecular characterization of a soybean polygalacturonase-inhibiting protein. Planta 195: 80–87. 7765794 10.1007/BF00206295

[37] Afzal AJ, Lightfoot DA (2007) Soybean disease resistance protein RHG1-LRR domain expressed, purified and refolded from Escherichia coli inclusion bodies: preparation for a functional analysis. Protein Expression Purif 53:346–355. 10.1016/j.pep.2006.12.017 17287130

[38] Taylor RJ, Secor GA (1988) An improved diffusion assay for quantifying the polygalacturonase content of *Erwinia* culture filtrates. Phytopathology 78:1101–1103.

[39] Sicilia F, Fernandez-Recio J, Caprari C, De Lorenzo G, Tsernoglou D, Cervone F, et al (2005) The polygalacturonase-inhibiting protein PGIP2 of Phaseolus vulgaris has evolved a mixed mode of inhibition of endopolygalacturonase PG1 of *Botrytis cinerea*. Plant Physiol 139: 1380–1388. 16244152 10.1104/pp.105.067546 PMC1283773

[40] Abu-Goukh AA, Labavitch JM (1983) The *in vivo* role of “Bartlett” pear fruit polygalacturonase inhibitors. Physiol Mol Plant Pathol 23:123–135.

[41] Mengiste T, Chen X, Salmeron J, Dietrich R (2003) The BOTRYTIS SUSCEPTIBLE1 gene encodes an *R2R3MYB* transcription factor protein that is required for biotic and abiotic stress responses in Arabidopsis. Plant Cell 15:2551–2565. 14555693 10.1105/tpc.014167 PMC280560

[42] Maulik A, Basu S (2013) Study of Q224K, V152G double mutation in bean PGIP2, an LRR protein for plant defense—An in silico approach. Proteins: Struct., Funct., Bioinf 81:852–862. 10.1002/prot.24243 23255146

[43] Abramovitch RB, Anderson JC, Martin GB (2006) Bacterial elicitation and evasion of plant innate immunity. Nat. Rev. Mol. Cell Biol 7: 601–611. 16936700 10.1038/nrm1984 PMC2842591

[44] Ramanathan V, Simpson CG, Thow G, Iannetta PPM, McNicol RJ, Williamson B (1997) cDNA cloning and expression of polygalacturonaseinhibiting proteins (PGIPs) from red raspberry (*Rubus idaeus*). J. Exp. Bot 48:1185–1193.

[45] Mattei B, Bernalda MS, Federici L, Roepstorff P, Cervone F, Boffi A (2001) Secondary structure and post-translational modifications of the leucine-rich repeat protein PGIP (polygalacturonase-inhibiting protein) from Phaseolus vulgaris. Biochemistry 40:569–576. 11148052 10.1021/bi0017632

[46] Wang R, Lu L, Pan X, Hu Z, Ling F, Yan Y, et al (2015) Functional analysis of *OsPGIP1* in rice sheath blight resistance. Plant Mol. Biol 87:181–191. 10.1007/s11103-014-0269-7 25488398

[47] Shirano Y, Kachroo P, Shah J, Klessig DF (2002) A gain-of-function mutation in an Arabidopsis Toll Interleukin1 Receptor–Nucleotide Binding Site–Leucine-Rich Repeat type R gene triggers defense responses and results in enhanced disease resistance. Plant Cell 14:3149–3162. 12468733 10.1105/tpc.005348 PMC151208

[48] Johnston DJ, Ramanathan V, Williamson B (1993) A protein from immature raspberry fruits which inhibits endopolygalacturonases from *Botrytis cinerea* and other micro-organisms. J. Exp. Bot 44:971–976.

[49] Sharrock KR, Labavitch JM (1994) Polygalacturonase inhibitors of *Bartlett pear* fruits: differential effects on *Botrytis cinerea* polygalacturonase isozymes, and influence on products of fungal hydrolysis of pear cell walls and on ethylene induction in cell culture. Physiol. Mol. Plant Pathol 45: 305–319.

[50] Leckie F, Mattei B, Capodicasa C, Hemmings A, Nuss L, Aracri B, et al (1999) The specificity of polygalacturonase‐inhibiting protein (PGIP): a single amino acid substitution in the solvent‐exposed β‐strand/β‐turn region of the leucine‐rich repeats (LRRs) confers a new recognition capability. EMBO J 18:2352–2363. 10228150 10.1093/emboj/18.9.2352 PMC1171318

[51] Cheong YH, Chang HS, Gupta R, Wang X, Zhu T, Luan S (2002) Transcriptional profiling reveals novel interactions between wounding, pathogen, abiotic stress, and hormonal responses in Arabidopsis. Plant Physiol 129: 661–677. 12068110 10.1104/pp.002857 PMC161692

[52] Lu L, Zhou F, Zhou Y, Fan X, Ye S, Wang L, et al (2012) Expression profile analysis of the polygalacturonase-inhibiting protein genes in rice and their responses to phytohormones and fungal infection. Plant cell rep 31:1173–1187. 10.1007/s00299-012-1239-7 22362377

[53] Ahsan N, Yoon HS, Jo J (2005) Molecular cloning of a *BcPGIP* cDNA from *Brassica campestris* and its expression to several stresses. Plant Sci 169: 1081–1089.

[54] Sathiyaraj G, Srinivasan S, Subramanium S, Kim YJ, Kim YJ, Kwon WS, et al (2010) Polygalacturonase inhibiting protein: isolation, developmental regulation and pathogen related expression in Panax ginseng CA Meyer. Mol. Biol. Rep 37:3445–3454. 10.1007/s11033-009-9936-1 19946753

[55] Ramonell KM, Somerville S (2002) The genomics parade of defense responses: to infinity and beyond. Curr. Opin. Plant Biol 5:291–294. 12179961 10.1016/s1369-5266(02)00266-2

[56] Johnson C, Boden E, Arias J (2003) Salicylic acid and NPR1 induce the recruitment of trans-activating TGA factors to a defense gene promoter in Arabidopsis. Plant Cell 15:1846–1858 12897257 10.1105/tpc.012211 PMC167174

[57] Grüner R, Strompen G, Pfitzner AJP, Pfitzner UM (2003) Salicylic acid and the hypersensitive response initiate distinct signal transduction pathways in tobacco that converge on the as-1-like element of the PR-1a promoter. Euro J Biochem 270: 4876–4886 10.1046/j.1432-1033.2003.03888.x 14653814

[58] D’ovidio R, Roberti S, Di Giovanni M, Capodicasa C, Melaragni M, Sella L, et al (2006) The characterization of the soybean polygalacturonase-inhibiting proteins (Pgip) gene family reveals that a single member is responsible for the activity detected in soybean tissues. Planta 224: 633–645. 16501991 10.1007/s00425-006-0235-y

[59] Szecsi A (1990) Analysis of Pectic Enzyme Zymograms of Fusarium Species. J. Phytopathol 130:188–196.

[60] Pettersen EF, Goddard TD, Huang CC, Couch GS, Greenblatt DM, Meng EC, et al (2004) UCSF Chimera—a visualization system for exploratory research and analysis. Journal of Computational Chemistry. 25:1605–1612. 15264254 10.1002/jcc.20084

[61] Veronese P, Nakagami H, Bluhm B, AbuQamar S, Chen XJ, Dietrich R, et al (2006) The membrane-anchored BOTRYTIS-INDUCED KINASE1 plays distinct roles in Arabidopsis resistance to necrotrophic and biotrophic pathogens. Plant Cell 18:257–273. 16339855 10.1105/tpc.105.035576 PMC1323497

[62] Zhu Y, Schluttenhoffer CM, Wang P, Fu F, Thimmapuram J, Zhu JK, et al (2014) CYCLIN-DEPENDENT KINASE8 Differentially Regulates Plant Immunity to Fungal Pathogens through Kinase-Dependent and-Independent Functions in Arabidopsis. Plant Cell 26: 4149–4170. 10.1105/tpc.114.128611 25281690 PMC4247566

