# Supplementary figures and images for: Molecular and Functional Characterization of a Polygalacturonase-Inhibiting Protein from Cynanchum komarovii That Confers Fungal Resistance in Arabidopsis
Source: PLoS One. 2016 Jan 11;11(1):e0146959. doi: 10.1371/journal.pone.0146959 (PMC4709088; doi:10.1371/journal.pone.0146959)

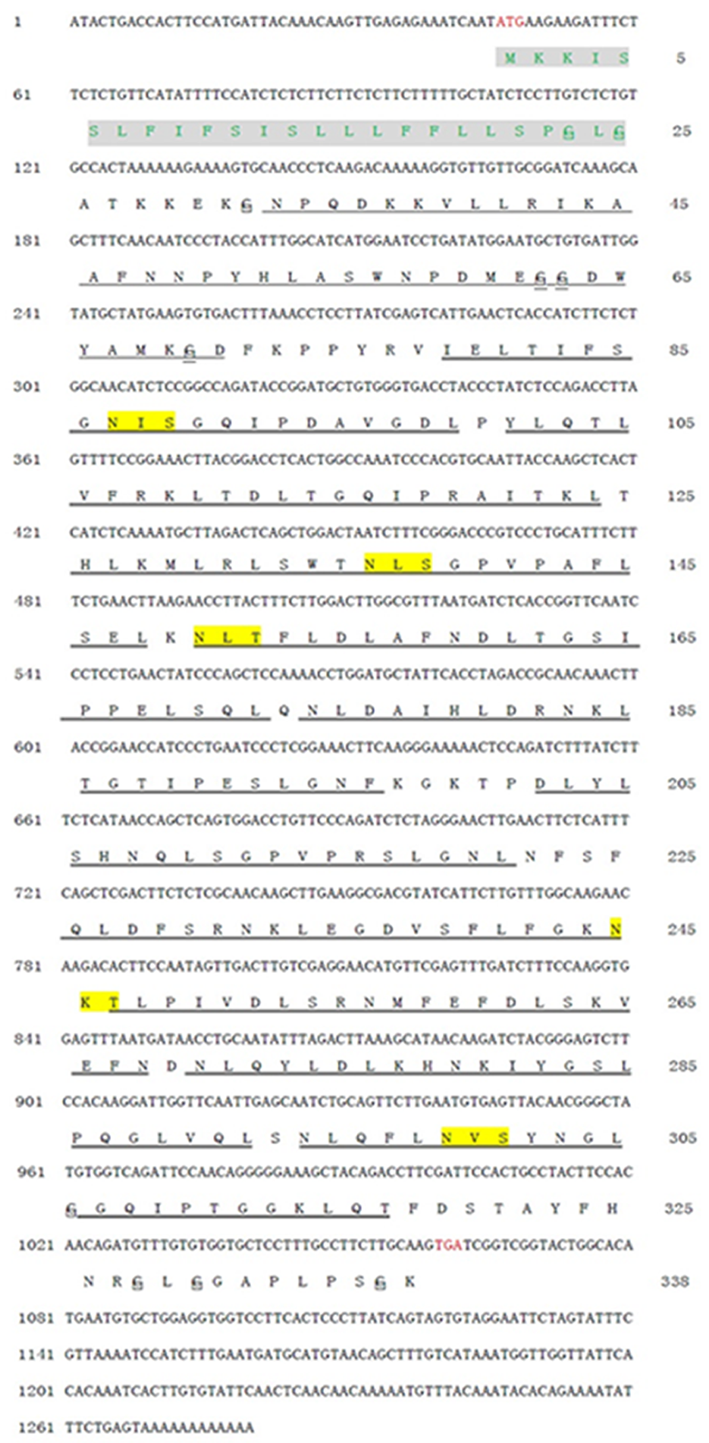

Supplement: S1 Fig — The signal peptide and N-glycosylation sites are highlighted in gray and yellow, respectively. LRRNT-2 and LRR domains are singly and doubly underlined, respectively. Cysteines are marked with diamonds. (TIF) [file pone.0146959.s001.tif]

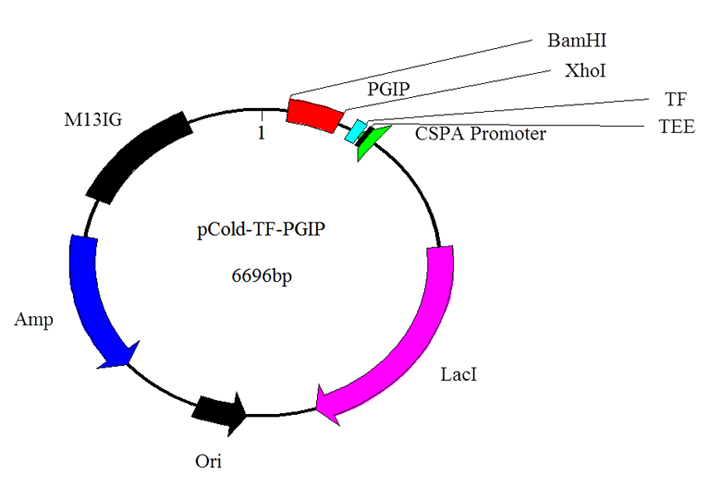

Supplement: S2 Fig — Homology models of CkPGIP1 (A), Botrytis cinerea (B), and Rhizoctonia solani polygalacturonase (C) are based on known structures of PvPGIP (1OGQ), and polygalacturonase from C. lupini (2IQ7), and Chondrostereum purpureum (1KCD). (TIF) [file pone.0146959.s002.tif]

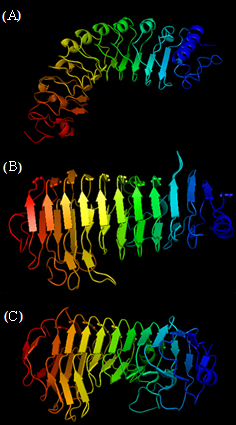

Supplement: S3 Fig — (TIF) [file pone.0146959.s003.tif]

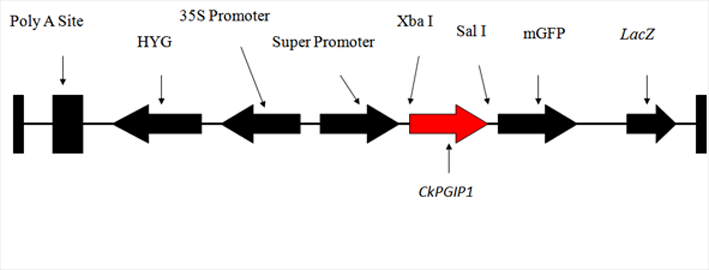

Supplement: S4 Fig — (TIF) [file pone.0146959.s004.tif]
