# Supplementary material for: Molecular and Functional Characterization of a Polygalacturonase-Inhibiting Protein from Cynanchum komarovii That Confers Fungal Resistance in Arabidopsis
Source: PLoS One. 2016 Jan 11;11(1):e0146959. doi: 10.1371/journal.pone.0146959 (PMC4709088; doi:10.1371/journal.pone.0146959)
Supplement: S1 Table — (A): before optimization;(B): after optimization. glob: the binding energy of the solution; aVdWa and rVdW: softened attractive and repulsive van der Waals energy; ACE: atomic contact energy; HB: hydrogen and disulfide bonds. (DOCX) [file pone.0146959.s006.docx]

| CkPGIP-BcPG1(B) CkPGIP-BcPG1(A) CkPGIP-RsPG1 (B) CkPGIP-RsPG1 (A) |
| --- |
| glob 7.24 -8.14 24.16 -8.82  aVdW -3.71 -37.77 -44.09 -39.45  rVdW 0.03 22.68 31.92 15.56  ACE 3.27 13.73 21.71 17.07  HB 0.00 -6.8 -5.65 -2.88 |

**Table S1. The comparison of all the changes about the complexes before and after optimization.**(**A**): before optimization;(**B**): after optimization. glob: the binding energy of the solution ; aVdWa and rVdW :softened attractive and repulsive van der Waals energy; ACE: atomic contact energy; HB: hydrogen and disulfide bonds.
